# Biomechanical comparison of noncontiguous cervical disc arthroplasty and noncontiguous cervical discectomy and fusion in the treatment of noncontinuous cervical degenerative disc disease: a finite element analysis

**DOI:** 10.1186/s13018-020-1549-3

**Published:** 2020-01-31

**Authors:** Xiangyao Sun, Siyuan Sun, Tongtong Zhang, Chao Kong, Wei Wang, Shibao Lu

**Affiliations:** 1 Department of Orthopaedics, Xuanwu Hospital Capital Medical University, Beijing, 100053 China; 2 National Clinical Research Center for Geriatric Diseases, Beijing, 100053 China; 3 Charité – Universitätsmedizin Berlin, Berlin, 113353 Germany; 4 Department of Interdisciplinary Life Science, Purdue University, West Lafayette, IN 47907 USA; 5 Department of Orthopaedics, ChuiYang Liu Hospital affiliated to Tsinghua University, Beijing, 100020 China

**Keywords:** Cervical degenerative disc disease, Anterior cervical discectomy and fusion, Intermediate segments, Cervical disc arthroplasty

## Abstract

**Background:**

Biomechanical characteristics of noncontinuous ACDF and noncontinuous CDA in the treatment of noncontinuous cervical degenerative disc disease were still unclear. The aim of this research is to compare the differences between these two kinds of treatment methods and to verify the effectiveness of Prodisc-C in noncontinuous CDA.

**Methods:**

Eight FEMs of the cervical spine (C2–C7) were built based on CT images of 8 mild CDDD volunteers. In the arthroplasty group, we inserted Prodisc-C at C3/4 and C5/6. In the fusion group, CoRoent® Contour and NuVasive® Helix ACP were implanted at C3/4 and C5/6. Initial loads of 75 N were used to simulate the head weight and muscle forces. The application of 1.0 N m moment on the top on the C2 vertebra was used to create motion in all directions. Statistical analyses were performed using STATA version 14.0 (Stata Corp LP, College Station, Texas, USA). Statistical significance was set at *P* < 0.05.

**Results:**

The IDPs in C2/3 (*P* < 0.001, *P* = 0.005, *P* < 0.001, *P* < 0.001), C4/5 (*P* < 0.001), and C6/7 (*P* < 0.001) of the intact group were significantly less than that in the fusion group in flexion, extension, lateral bending, and axial rotation, respectively. In addition, the IDPs in C2/3 (*P* < 0.001, *P* = 0.001, *P* < 0.001, *P* < 0.001), C4/5 (*P* < 0.001), and C6/7 (*P* < 0.001) of the arthroplasty group were significantly less than that in the fusion group in flexion, extension, lateral bending, and axial rotation, respectively. Contact forces of facet joints in C2/3 (*P* = 0.010) in the arthroplasty group was significantly less than that in the intact group. Contact forces of facet joints in C2/3 (*P* < 0.001), C4/5 (*P* < 0.001), and C6/7 (*P* < 0.001) in the arthroplasty group was significantly less than that in the fusion group. Contact forces of facet joints in C2/3 (*P* < 0.001), C4/5 (*P* < 0.001), and C6/7 (*P* < 0.001) in the intact group were significantly less than that in the fusion group.

**Conclusions:**

Noncontinuous CDA could preserve IDP and facet joint forces at the adjacent and intermediate levels to maintain the kinematics of cervical spine near preoperative values. However, noncontinuous ACDF would increase degenerative risks at adjacent and intermediate levels. In addition, the application of Prodisc-C in noncontinuous CAD may have more advantages than that of Prestige LP.

## Introduction

Noncontiguous cervical degenerative disc disease (CDDD) is defined as cervical myelopathy or radiculopathy caused by two noncontiguous degenerative intervertebral discs with one normal intermediate segment (IS) [1]. Anterior cervical discectomy and fusion (ACDF) has been an accepted treatment method for degenerative cervical disc disease to alleviate cervical myelopathy or radiculopathy [2]. Previous studies indicated that the treatment effect of ACDF was excellent with over 90% of patients whose reduced movement functions were improved [3]. Long segmental anterior fusion, which included the normal intermediate segments (IS), was always used to treat noncontiguous CDDD in order to decrease the stress from fusion structures on IS and avoid the adjacent segment degeneration (ASD) in IS [4–6]. In general, long segmental anterior fusion was associated with high risk of pseudarthrosis, persistent postoperative dysphagia, nonunion, and ASD [4–6]. It has been reported that the preservation of IS could ameliorate postoperative outcomes [7]. However, most of the studies preserved the IS with noncontinuous ACDF, which would bring more additive stress from the fused levels and then cause hypermobility on IS; all of these would cause the acceleration of ASD [8].

Compared with ACDF, cervical disc arthroplasty (CDA) can preserve the motion at the operated level and theoretically alleviate ASD [9]. Previous studies reported that multilevel CDA could achieve better clinical outcomes compared with one-level CDA, even though the surgical techniques of multilevel CDA were more difficult and the inclusion criteria were stricter [10, 11]. Furthermore, Wu et al. [8] reported that noncontinuous CDA could reduce the biomechanical impact on the IS compared with noncontinuous fusion. However, they only built a standard set of models for analysis, lacking a statistical comparison of multiple patient models. In addition, most studies have discussed the effectiveness of Prestige LP in noncontinuous CDA, but the use of Prodisc-C in this kind of operation has not been fully discussed [1, 8, 12, 13]. Therefore, the aim of this research is to analyze the biomechanical characteristics of noncontinuous ACDF and noncontinuous CDA based on multiple patient models, comparing the differences between these two kinds of treatment methods, and to verify the effectiveness of Prodisc-C in noncontinuous CDA.

## Methods

### Geometry models

The finite element models (FEMs) of the cervical spine (C2–C7) were built according to the method reported by Rong et al. [14]. The models were constructed based on the CT images (a 0.75 mm thickness and a 0.69-mm interval, SOMATOM Definition AS+, Siemens, Germany) of 8 mild CDDD volunteers (4 male and 4 females). A commercial software Mimics 17.0 (Materialize Inc, Leuven, Belgium) was used to transform the CT images into the solid models of the C2–C7 vertebrae and output STL files. The reconstructed models were then imported into Geomagic Studio 12.0 (3D System Corporation, Rock Hill, SC, USA) to change the models into physical structures.

The devices, CoRoent® Contour (NuVasive, Inc., San Diego, CA, USA), NuVasive® Helix ACP (NuVasive, Inc. San Diego, CA, USA), and Prodisc-C (Synthes, Inc., West Chester, PA, USA ), were included in this study. CoRoent® Contour was 17 mm long, 14 mm wide, 6 mm high, and 7° lordotic. The dimensions (width, length, and thickness) of NuVasive® Helix ACP were 16 mm, 24 mm, and 2.4 mm. The diameter and length of self-tapping screws were 4.5 mm and 14 mm, respectively. Prodisc-C was 16 mm long, 15 mm wide, and 6 mm high. All the FEMs of implants were made in Solidworks 2016 (Dassault Systèmes, MA, USA).

**Fig. 1:**
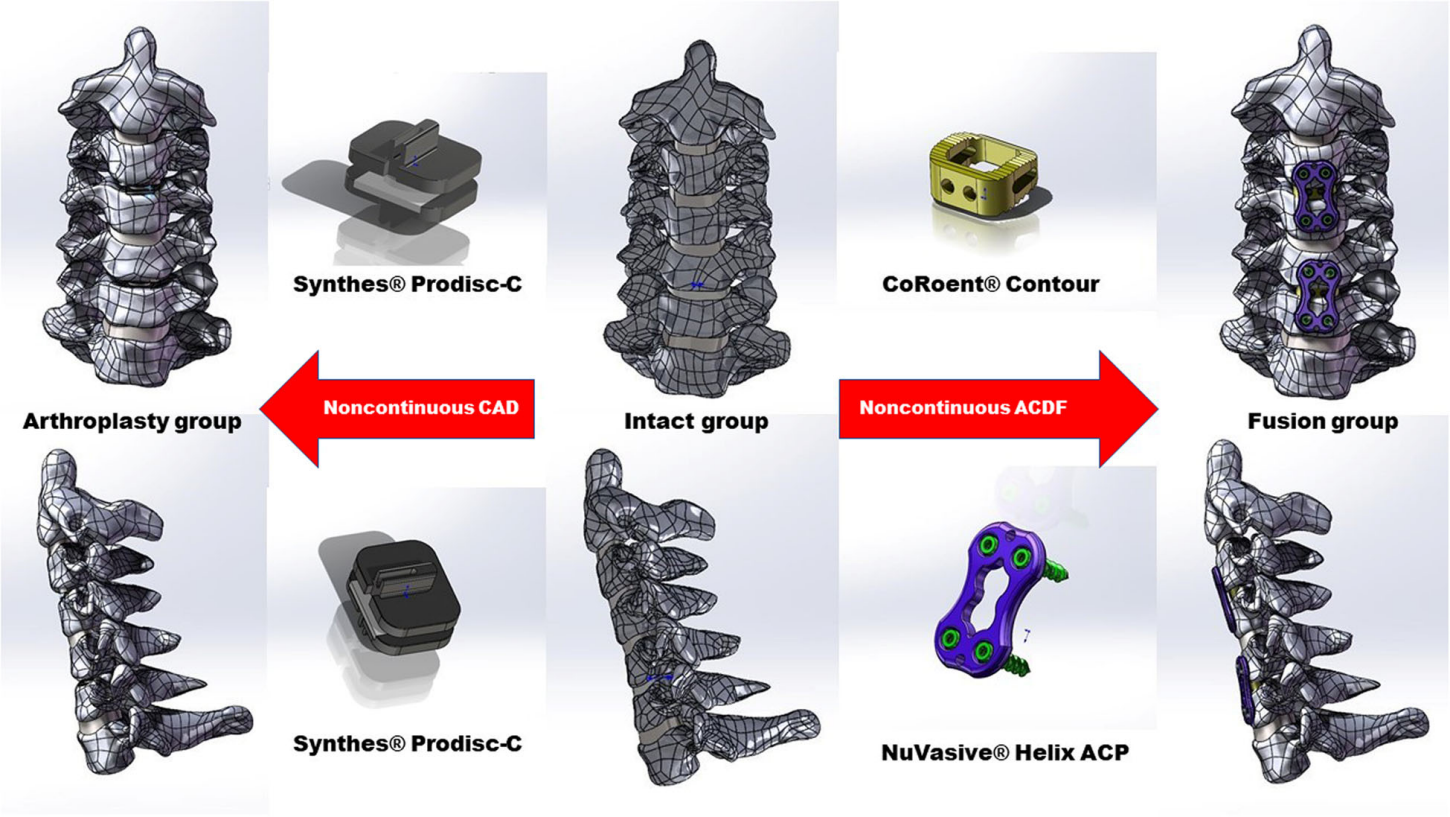
FEMs of the intact group, arthroplasty group, and fusion group

The implants and the cervical vertebra models were assembled in the software mentioned above. Considering the C3/4 and C5/6 were the most frequently discussed levels in previous studies, these two levels were chosen as the implanted levels in our study [1, 8, 12]. In the arthroplasty group, we inserted Prodisc-C at C3/4 and C5/6 after removing the anterior longitudinal ligament (ALL), the posterior longitudinal ligament (PLL), and intervertebral discs at the corresponding locations. In the fusion group, CoRoent® Contour and NuVasive® Helix ACP were implanted at C3/4 and C5/6 after removing the relevant soft tissues (Fig. 1).

Next, the high-quality FE meshes of the models were developed in Hypermesh 12.0 (Altair, Troy, MI, USA). Finally, the Models were imported into ABAQUS 6.13 (Dassault Systems Corporation, MA, USA) to set the material properties, boundary conditions, loading modes, and perform analysis.

### Material properties

In these FEMs, the cortical bone and vertebral endplates were 0.4-mm thick shells [15]. The ratio of annulus fibrosus and nucleus pulposus in the intervertebral disc was 6:4; annulus fibers, which comprised 19% of the total annulus fibrosus volume, were developed with an inclination (15 to 30°) to the transverse plane [15]. The distance of the upper and lower facet articular surfaces was 0.5 mm; the facet articular surfaces were covered with articular cartilage layer; the surface to surface contact was set to nonlinear [14]. The five intervertebral ligaments, the ALL, the PLL, the ligamentum flavum (LF), the interspinous ligaments (ILs), and capsular ligaments (CLs), were modeled as tension-only truss elements and attached to the corresponding vertebrae. Table 1 showed the material properties and mesh types of FEMs.

**Table 1:** Material Properties and mesh types of cervical spine and implants

Cervical component	Young’s modulus (MPa)	Poisson ratio	Cross-sectional area (mm^2^)	Element type
Cortical bone	12000	0.29	-	C3D4
Cancellous bone	100	0.29	-	C3D4
Endplate	1200	0.29	-	C3D4
Cartilage	10.4	0.4	-	C3D4
Annulus ground substance	3.4	0.4	-	C3D4
Annulus fibers	450	0.45	-	T3D2
Nucleus pulposus	1	0.49	-	C3D4
ALL	30	0.3	12	T3D2
PLL	20	0.3	45	T3D2
LF	1.5	0.3	5	T3D2
IL	1.5	0.3	13	T3D2
SL	1.5	0.3	13.1	T3D2
CL	10	0.3	14	T3D2
Ti6Al4V	114.000	0.35	-	C3D4
PEEK	3400	0.4	-	C3D4

*ALL* anterior longitudinal ligament, *CL* capsular ligament, *IL* interspinous ligament, *LF* ligament flavum, *PLL* posterior longitudinal ligament, *SL* supraspinous ligament, *C3D4* tetrahedron, *T3D2* truss, tension only

### Experimental condition

Fixed inferior surface of C7 vertebra and a tie connection between adjacent endplates and intervertebral discs were used to simulate the boundary condition in vitro experiments [16]. The cancellous bone that fills the CoRoent® Contour was set to frictionless; the simulation of the rigidly fusion between graft-vertebrae interfaces and full osseointegration between implant and vertebrae was carried out by the application of a tie constraint; the frictionless contact was applied to the implant-implant interfaces of Prodisc-C [17].

Initial loads of 75 N were used to simulate the head weight and muscle forces. The application of 1.0 N m moment on the top on the C2 vertebra was used to produce the motion of flexion, extension, lateral bending, and axial rotation. The validation of the effectiveness of our FEMs was carried out by comparing the range of motion of the segments in our FEMs with the published data. Considering the patients would attempt to move their cervical spine in a range of motion (ROM) similar to their preoperative conditions, the displacement-control test protocol was used in our subsequent evaluations.

### Statistical analysis

STATA version 14.0 (Stata Corp LP, College Station, Texas, USA) was used to carry out the statistical analysis. Continuous variables were presented as mean ± standard deviations (SD). Normality of the continuous data was analyzed by Kolmogorov-Smirnov test. Normally distributed values were tested using one-way analysis of variance (ANOVA) or Student’s *t* test. Kruskal-Wallis test was used to analyze skew distributed values. A *P* value < 0.05 was statistically significant.

## Results

### Validation of the intact FEMs

**Fig. 2:**
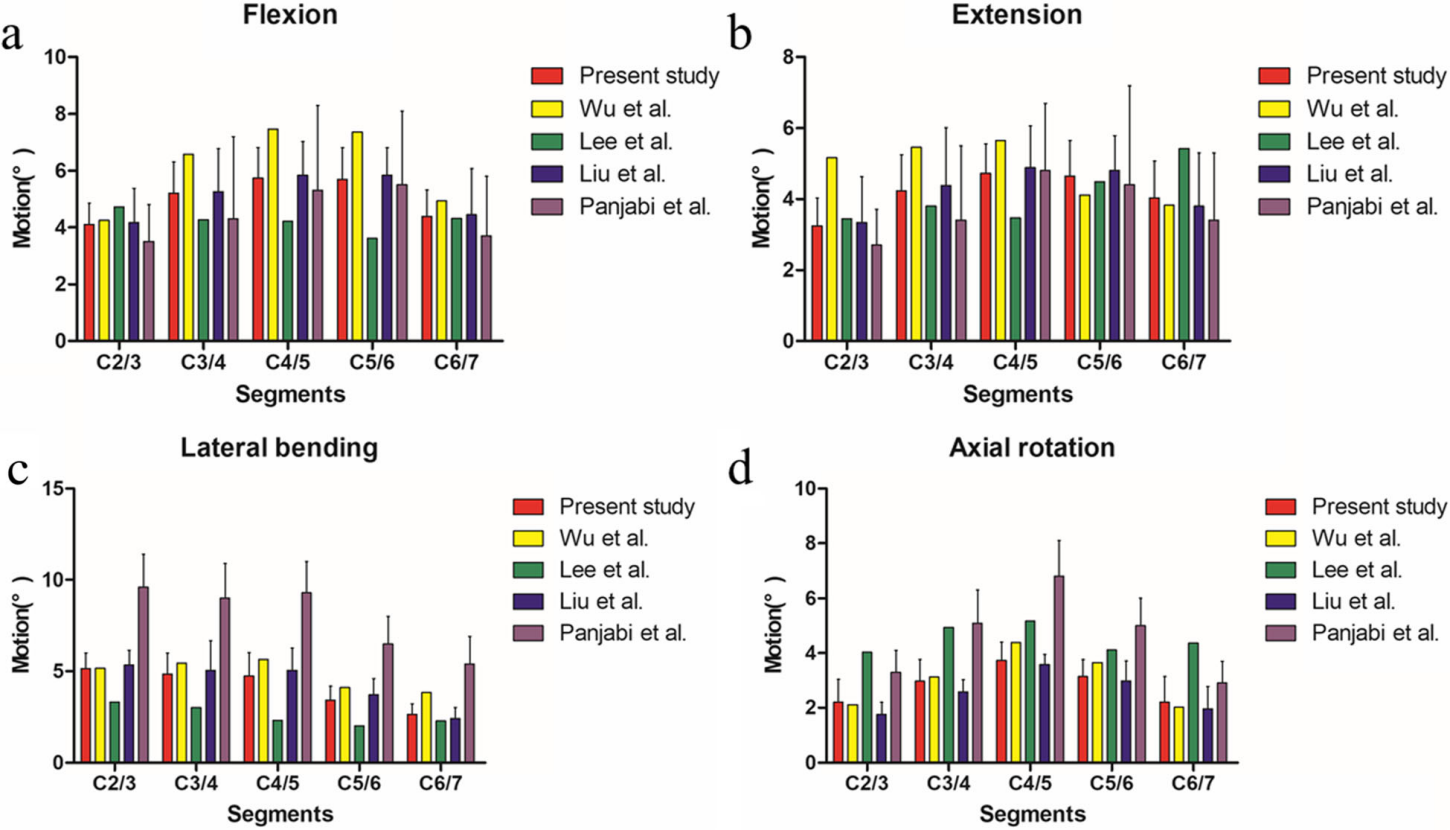
**a**–**d** ROMs of FEMs are validated by previous studies

ROMs of our FEMs in flexion-extension, lateral bending, and axial rotation were compared with the data from previous studies [8, 18–20]. The ROMs of the intact FEMs at C2/3, C3/4, C4/5, C5/6, and C6/7 were 4.11° ± 0.75°, 5.22° ± 1.10°, 5.74° ± 1.08°, 5.70° ± 1.11°, and 4.39° ± 0.94°, respectively, in flexion; 3.24° ± 0.79°, 4.23° ± 1.03°, 4.65° ± 1.01°, and 4.04° ± 1.03°, respectively, in extension; 5.15° ± 0.85°, 4.84° ± 1.15°, 4.73° ± 1.29°, 3.42° ± 0.77°, and 2.63° ± 0.58°, respectively, in lateral bending; and 2.04° ± 0.83°, 2.97° ± 0.79°, 3.73° ± 0.67°, 3.14° ± 0.62°, and 2.20° ± 0.93°, respectively, axial rotation (Fig. 2). The segmental ROMs of our FEMs were in good agreement with the published data.

### ROM at different levels

**Fig. 3:**
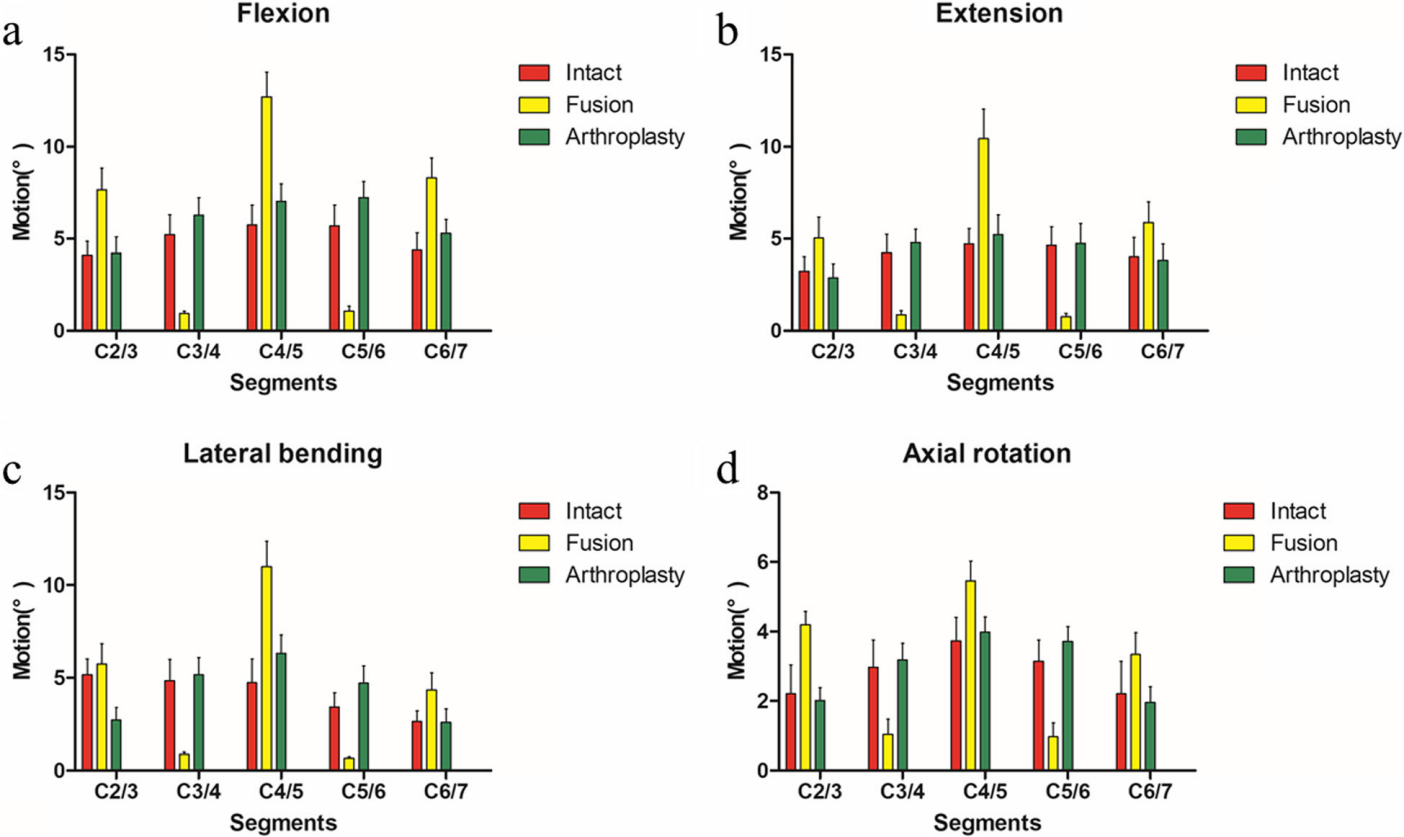
ROMs of FEMs under different motion states. **a** Flexion, **b** extension, **c** lateral bending, and **d** axial rotation

In comparison of ROMs at different intervertebral levels between the arthroplasty group and intact group, the results showed the ROMs in flexion of C4/5 (*P* = 0.032) and C6/7 (*P* = 0.013) in the arthroplasty group were significantly higher than that in the intact group; the ROM in lateral bending of C2/3 (*P* < 0.001) in the arthroplasty group was significantly less than that in the intact group; however, the ROM in lateral bending of C5/6 (*P* = 0.014) in the arthroplasty group was significantly more than that in the intact group. In comparison of ROMs at different intervertebral levels between the intact group and fusion group, the results showed the ROMs of C2/3 (*P* < 0.001, *P* = 0.004, *P* < 0.001), C4/5 (*P* < 0.001), and C6/7 (*P* < 0.001, *P* = 0.006, *P* = 0.002) in the intact group were significantly less than that in the fusion group in flexion, extension, and axial rotation, respectively; the ROMs of C3/4 (*P* < 0.001) and C5/6 (*P* < 0.001) in the intact group were significantly higher than that in the fusion group in flexion, extension, lateral bending, and axial rotation, respectively; the ROMs in lateral bending of C4/5 (*P* < 0.001) and C6/7 (*P* = 0.002) were significantly less than that in the fusion group. In comparison of ROMs at different intervertebral levels between the arthroplasty group and fusion group, the results showed the ROMs of C2/3 (*P* ≤ 0.001), C4/5 (*P* < 0.001), and C6/7 (*P* < 0.001, *P* = 0.002, *P* = 0.002, *P* = 0.002) in the arthroplasty group were significantly less than that in the fusion group in flexion, extension, lateral bending, and axial rotation, respectively; the ROMs of C3/4 (*P* < 0.001) and C5/6 (*P* < 0.001) in the arthroplasty group were significantly higher than that in the fusion group in flexion, extension, lateral bending, and axial rotation, respectively. In other situations, there was no significant difference in ROMs between each of the two groups (Table 2, Fig. 3).

**Table 2:** Comparison of ROMs at different intervertebral levels

Motion (°)	Segments	Models			*P* values		
		Intact	Arthroplasty	Fusion	Intact vs arthroplasty	Intact vs fusion	Arthroplasty vs fusion
Flexion							
	C2/3	4.10 ± 0.75	4.22 ± 0.88	7.67 ± 1.17	0.791	< 0.001	< 0.001
	C3/4	5.22 ± 1.09	6.27 ± 0.95	0.94 ± 0.14	0.075	< 0.001	< 0.001
	C4/5	5.74 ± 1.08	7.03 ± 0.94	12.72 ± 1.33	0.032	< 0.001	< 0.001
	C5/6	5.70 ± 1.11	7.23 ± 0.88	1.08 ± 0.26	0.013	< 0.001	< 0.001
	C6/7	4.39 ± 0.94	5.29 ± 0.75	8.31 ± 1.07	0.067	< 0.001	< 0.001
Extension							
	C2/3	3.24 ± 0.79	2.88 ± 0.77	5.03 ± 1.13	0.398	0.004	0.001
	C3/4	4.23 ± 1.03	4.79 ± 0.72	0.87 ± 0.23	0.256	< 0.001	< 0.001
	C4/5	4.72 ± 0.83	5.23 ± 1.06	10.45 ± 1.60	0.338	< 0.001	< 0.001
	C5/6	4.65 ± 1.01	4.74 ± 1.09	0.77 ± 0.18	0.866	< 0.001	< 0.001
	C6/7	4.04 ± 1.03	3.83 ± 0.88	5.88 ± 1.11	0.688	0.006	0.002
Lateral bending							
	C2/3	5.15 ± 0.85	2.71 ± 0.69	5.75 ± 1.10	< 0.001	0.277	< 0.001
	C3/4	4.84 ± 1.15	5.16 ± 0.94	0.86 ± 0.11	0.582	< 0.001	< 0.001
	C4/5	4.73 ± 1.29	6.31 ± 1.00	10.99 ± 1.38	0.971	< 0.001	< 0.001
	C5/6	3.42 ± 0.77	4.70 ± 0.95	0.66 ± 0.08	0.014	< 0.001	< 0.001
	C6/7	2.63 ± 0.58	2.58 ± 0.74	4.33 ± 0.93	0.902	0.002	0.002
Axial rotation							
	C2/3	2.20 ± 0.83	2.01 ± 0.34	4.19 ± 0.38	0.574	< 0.001	< 0.001
	C3/4	2.97 ± 0.79	3.18 ± 0.48	1.03 ± 0.45	0.562	< 0.001	< 0.001
	C4/5	3.73 ± 0.67	3.99 ± 0.43	5.45 ± 0.57	0.407	< 0.001	< 0.001
	C5/6	3.14 ± 0.62	3.71 ± 0.43	0.96 ± 0.40	0.065	< 0.001	< 0.001
	C6/7	2.20 ± 0.93	1.95 ± 0.45	3.34 ± 0.63	0.532	0.019	0.002

*ROM* range of motion

### Intervertebral disc pressures (IDPs) at adjacent levels and in ISs

**Fig. 4:**
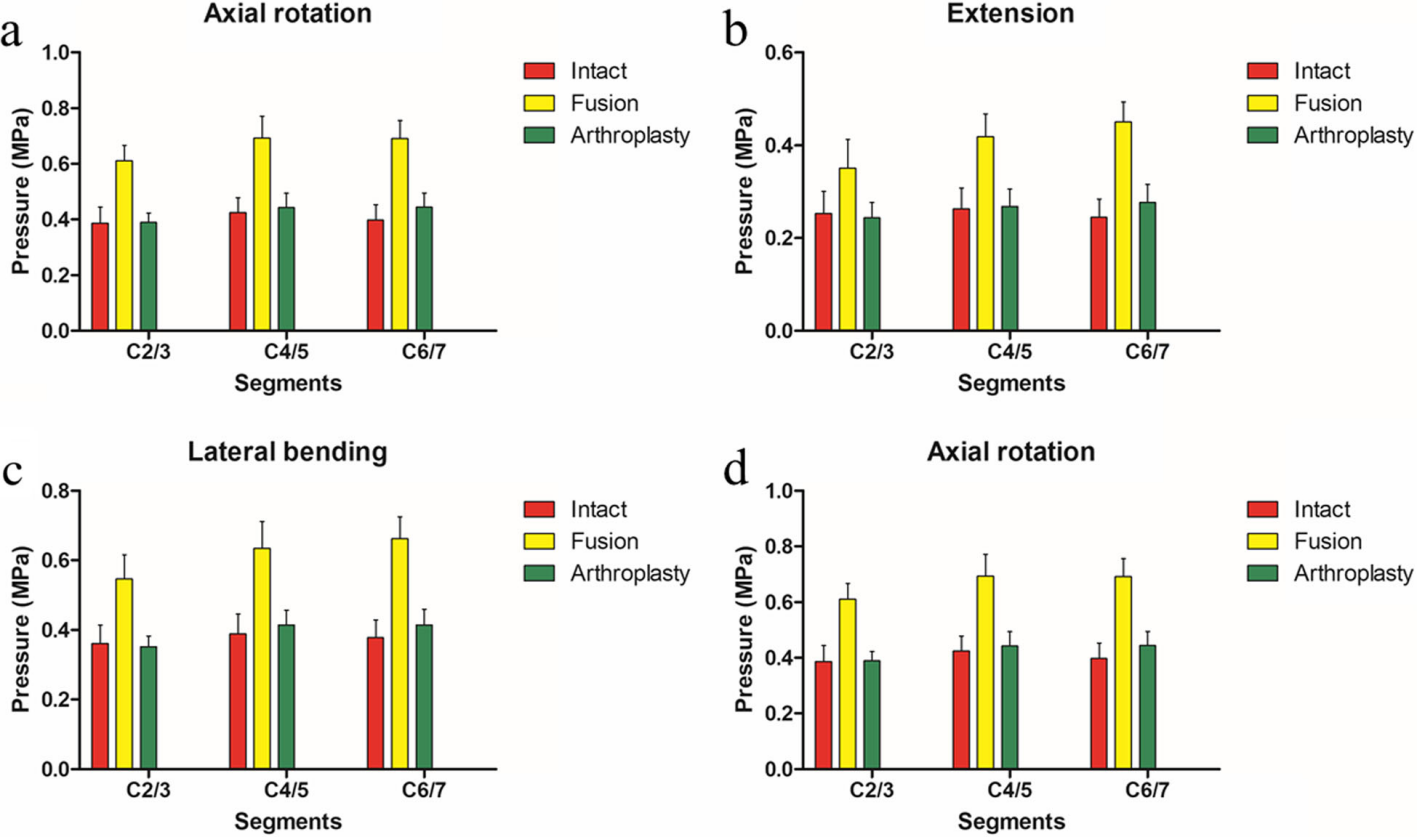
IDPs of FEMs under different motion states. **a** Flexion, **b** extension, **c** lateral bending, and **d** axial rotation

Table 3 showed that there was no significant difference in IDPs between the arthroplasty group and intact group in all situations. However, the IDPs in C2/3 (*P* < 0.001, *P* = 0.005, *P* < 0.001, *P* < 0.001), C4/5 (*P* < 0.001), and C6/7 (*P* < 0.001) of the intact group were significantly less than that in the fusion group in flexion, extension, lateral bending, and axial rotation, respectively. In addition, the IDPs in C2/3 (*P* < 0.001, *P* = 0.001, *P* < 0.001, *P* < 0.001), C4/5 (*P* < 0.001), and C6/7 (*P* < 0.001) of the arthroplasty group were significantly less than that in the fusion group in flexion, extension, lateral bending, and axial rotation, respectively. In other situations, there was no significant difference in IDPs between each of the two groups (Fig. 4).

**Table 3:** Comparison of average pressures in intervertebral discs at different intervertebral levels

Pressure (MPa)	Segments	Models			*P* values		
		Intact	Arthroplasty	Fusion	Intact vs arthroplasty	Intact vs fusion	Arthroplasty vs fusion
Flexion							
	C2/3	0.21 ± 0.05	0.23 ± 0.03	0.34 ± 0.06	0.440	< 0.001	< 0.001
	C4/5	0.26 ± 0.05	0.27 ± 0.04	0.41 ± 0.06	0.704	< 0.001	< 0.001
	C6/7	0.24 ± 0.04	0.27 ± 0.04	0.43 ± 0.05	0.138	< 0.001	< 0.001
Extension							
	C2/3	0.25 ± 0.05	0.24 ± 0.03	0.35 ± 0.06	0.690	0.005	0.001
	C4/5	0.26 ± 0.045	0.27 ± 0.04	0.42 ± 0.05	0.815	< 0.001	< 0.001
	C6/7	0.24 ± 0.04	0.28 ± 0.04	0.45 ± 0.04	0.151	< 0.001	< 0.001
Lateral bending							
	C2/3	0.36 ± 0.05	0.35 ± 0.03	0.55 ± 0.07	0.701	< 0.001	< 0.001
	C4/5	0.39 ± 0.06	0.41 ± 0.04	0.63 ± 0.08	0.350	< 0.001	< 0.001
	C6/7	0.38 ± 0.05	0.41 ± 0.04	0.66 ± 0.06	0.176	< 0.001	< 0.001
Axial rotation							
	C2/3	0.39 ± 0.06	0.39 ± 0.03	0.61 ± 0.06	0.896	< 0.001	< 0.001
	C4/5	0.42 ± 0.05	0.44 ± 0.05	0.69 ± 0.08	0.518	< 0.001	< 0.001
	C6/7	0.40 ± 0.06	0.44 ± 0.05	0.69 ± 0.06	0.123	< 0.001	< 0.001

### Contact forces of facet joints at adjacent levels and in ISs

**Fig. 5:**
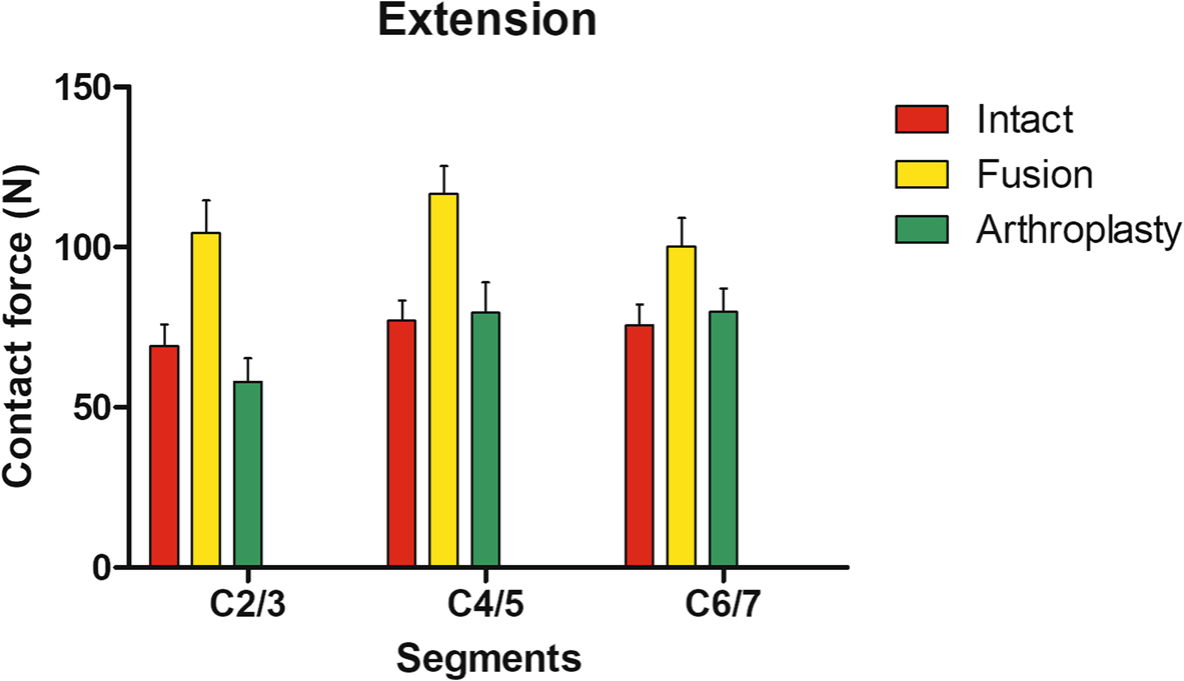
The facet contact forces of FEMs at extension

Contact forces of facet joints in C2/3 (*P* = 0.010) in the arthroplasty group was significantly less than that in the intact group in extension. Contact forces of facet joints in C2/3 (*P* < 0.001), C4/5 (*P* < 0.001), and C6/7 (*P* < 0.001) in the arthroplasty group was significantly less than that in the fusion group. In extension, contact forces of facet joints in C2/3 (*P* < 0.001), C4/5 (*P* < 0.001), and C6/7 (*P* < 0.001) in the intact group were significantly less than that in the fusion group (Table 4, Fig. 5).

**Table 4:** Comparison of average forces in facet joints at different intervertebral levels in extension

Segments	Contact forces in models (*N*)			*P* values		
	Intact	Arthroplasty	Fusion	Intact vs arthroplasty	Intact vs fusion	Arthroplasty vs fusion
C2/3	69.25 ± 6.73	57.95 ± 7.38	104.42 ± 10.10	0.010	< 0.001	< 0.001
C4/5	77.03 ± 6.46	79.54 ± 9.51	116.64 ± 8.72	0.572	< 0.001	< 0.001
C6/7	75.70 ± 6.46	79.86 ± 7.37	100.16 ± 8.91	0.281	< 0.001	< 0.001

## Discussion

The optimal surgical treatment of multilevel CDDD is still controversial [21]. Clinical data on the surgical protocol for noncontinuous CDDD are limited [8]. ACDF is widely used to treat multilevel CDDD [22]. However, several studies reported that ACDF could result in certain complications [9, 23–28]. It has been reported that the incidences of complications in different levels of fusion are variable: the rates of internal fixation failures in one to four-level fusion are 20%, 36%, 71%, and 80%, respectively [23]; the incidences of reoperation in one to four-level fusion are 5.8%, 6.5%, 8%, and 16.8%, respectively [27]; in addition, the incidences of ASD in single-level fusion and multilevel fusion are 13.2% and 32.1%, respectively [29]. Several studies indicated that patients could benefit from CDA over ACDF in clinical scoring systems and reoperation rates in long-term follow-up [30, 31]. Meta-analysis showed that the outcomes of two-level CDA were better than the outcomes after two-level ACDF [32]. All these implied that multilevel CDA might be reasonable. Lu et al. [31] reported that there was a significant difference in diagnosis, implying that while CDA was more likely to be applied to the treatment of cervical disc herniation, it was less likely to be applied to the treatment of cervical myelopathy, cervical stenosis, and cervical spondylosis than ACDF [31]. Therefore, biomechanical studies are needed to prove the effectiveness of various surgical treatment methods.

Comparison of ROMs at different intervertebral levels between each of the two groups showed that noncontinuous CDA could significantly increase the ROM in flexion at implanted levels and lower adjacent levels. This implied that noncontinuous CDA requires high biomechanical properties of the lower adjacent intervertebral discs. Patients with intervertebral disc degeneration at the lower adjacent segments would not be eligible for this kind of treatment. Results in our study showed that noncontinuous CDA could increase ROMs of lateral bending at implanted levels. In contrast, it would limit the ROMs of lateral bending in the upper adjacent segments. This might be explained by the relatively lower center of rotations (CORs) in Prodisc-C artificial discs [33]. The limitation of lateral bending in the upper adjacent segments could preserve the facet joints from degeneration, considering larger lateral bending ROM could increase facet joint forces [34]. This might be one of the reasons why CDA could reduce the stress of facet joints.

The results of comparison of IDP between each of the two groups in our study showed that noncontinuous CDA could preserve IDP at the adjacent and intermediate levels to maintain the kinematics of cervical spine near preoperative values. However, Wu et al. [8] stated that noncontinuous CDA could slightly increase the IDPs at the superior, intermediate, and inferior adjacent levels. The possible explanation might be that they only discussed one standard symmetric FEM to draw conclusion without statistical analysis; our study analyzed eight FEMs and ran a statistical analysis to compare the differences between each of the two groups. Therefore, our results might be more convincing. In addition, the artificial intervertebral discs we used in this study were Prodisc-C artificial discs, which were different with Prestige LP artificial discs in previous studies [1, 8, 12, 13]. Previous studies reported that design concepts of artificial discs could reveal different biomechanical characteristics for the treatment of CDDD [20]. The design of Prestige LP is metal-on-metal joint without polymercore. Previous study reported that the posteriorly positioned metal-on-metal joint of Prestige LP could, even in flexion, posteriorly impose a high stress level [2]. However, Prodisc-C has a polyethylene core with much higher modulus. Compared with Prestige LP, its stress transmission could be less and its distribution of loads could be more even [2]. Therefore, the application of Prodisc-C in noncontinuous CAD may have more advantages. However, noncontinuous ACDF would significantly increase IPD at adjacent and intermediate levels. This was mainly related to the fact that ACDF reduced the ROM of surgical segments, while increased the compensation of adjacent segments, resulting in a decreased stress buffering capacity and an increased stress concentration [8].

Facet degeneration has been proved to be most important cause of neck pain [16]. Progression of facet degeneration could result from too large loading [35]. Fusion cervical model needs bigger bending moment than the intact cervical model to reach a reasonable ROM. It was reported that bigger bending moment could increase facet joint forces and segmental rotation in all adjacent segments of the fusion model [34]. Similarly, our results showed that noncontinuous ACDF would increase the contact forces of facet joints at the adjacent and intermediate levels. Therefore, our study proved that the degeneration of facet joints could be deteriorated by noncontinuous ACDF. Lee et al. [33] stated that artificial discs could increase the stresses sustained by the facet joints. The ligamentous FEM used in their study could cause several limitations in their conclusions. They found that contact forces of facet joints increased by 107% with the Prodisc-C model, which was a surprising phenomenon. Our study constructed eight FEMs based on CT images and carried out the experiment based on displacement-control test protocol, which could handle the limitations mentioned above. The results in our study showed that noncontinuous CDA could reduce facet joint forces to reach the value of intact cervical spine. It could even make the facet joint forces at adjacent levels less than that in intact cervical spine. This might be explained by the intervertebral distractive effect of Prodisc-C, which could distribute the stress of facet joints [2].

Recent studies showed that hybrid surgery (HS), which incorporated CDA at the mobile segment with ACDF at the spondylotic segment, could preserve the mobility of cervical spine to produce satisfactory clinical outcomes and reducing ASD [10, 11, 36–38]. Considering multilevel CDDD can have different degenerative status at each level, HS may not always be appropriate to treat this kind of disease [12]. Most of the studies on HS focused on the treatment of continuous CDDD; however, there is a lack of study on the treatment of noncontiguous CDDD [8]. Previous study reported that noncontinuous HS could cause the collapse of IS [3]. Therefore, the use of HS in the treatment of noncontinuous CDDD might be risky.

There are several limitations in our study. First, the data discussed in this study depend on eight FEMs. The biomechanics of our FEMs may not completely simulate the pathology of CDDD in vivo, considering the number of ISs may be more than one and the ISs may not always be located in C3/4. Second, the elastic modulus and Poisson’s ratio of degenerative cervical soft tissues have not been reported in the previous studies. Therefore, the simulation of real CDDD via the analysis of FEMs is very difficult. However, our study is the first to conduct statistical analysis of multiple models, which can improve the accuracy of the results. Even so, our results can only provide an estimate of the trend rather than the actual value in the real situations. Third, patients may belong to different cervical sagittal classifications. Even though we have constructed eight FEMs to reduce the influence of cervical sagittal classifications on our results, the biomechanical characteristics of cervical sagittal classifications, and their influence on surgical treatment still needs to be discussed separately in the future researches.

## Conclusion

The analysis of FEMs shows that the overall therapeutic effect of noncontinuous CDA is better than that of noncontinuous ACDF in the treatment of noncontinuous CDDD. Noncontinuous CDA requires high biomechanical properties of the lower adjacent intervertebral discs. Patients with intervertebral disc degeneration at the lower adjacent segments would not be eligible for this kind of treatment. Noncontinuous CDA could preserve IDP and facet joint forces at the adjacent and intermediate levels to maintain the kinematics of cervical spine near preoperative values. However, noncontinuous ACDF would increase degenerative risks at adjacent and intermediate levels. In addition, the application of Prodisc-C in noncontinuous CAD may have more advantages than that of Prestige LP. A large number of in vivo studies are still needed to draw more reliable conclusions.

## Data Availability

Please contact author for data requests.

## Abbreviations

CDDD: Cervical degenerative disc disease
ACDF: Anterior cervical discectomy and fusion
IS: Intermediate segment
ASD: Adjacent segment degeneration
CDA: Cervical disc arthroplasty
FEMs: Finite element models
ALL: Anterior longitudinal ligament
PLL: The posterior longitudinal ligament
LF: The ligamentum flavum
IL: The interspinous ligament
CL: Capsular ligament
ROM: Range of motion
SD: Standard deviation
ANOVA: One-way analysis of variance
IDP: Intervertebral disc pressure
CORs: Center of rotations
HS: Hybrid surgery
